# Proliferation/quiescence: the controversial "aller-retour"

**DOI:** 10.1186/1747-1028-6-10

**Published:** 2011-05-09

**Authors:** Bertrand Daignan-Fornier, Isabelle Sagot

**Affiliations:** 1 Institut de Biochimie et Génétique Cellulaires, Université Bordeaux, Bordeaux Cedex, France; 2 CNRS - UMR5095 Bordeaux, France

## Abstract

The vast majority of cells, from prokaryotes up to vertebrate organisms, spend most of their time in quiescence, a state defined as a temporary and reversible absence of proliferation. Establishing the quiescent state while maintaining the capacity to re-enter the proliferation cycle are critical for cell survival and must be tightly orchestrated to avoid pathological proliferation. Hence, studying the biology of quiescent cells is an exciting research field. Taking advantage of technical progress in genomic, transcriptomic and metabolomic, the nature of transitions between proliferation and quiescence have been recently re-visited in budding yeast. Together with new findings in cell biology, these studies resuscitate an old demon in the field: the controversial existence of a "quiescence program".

## Introduction

**Figure 1:**
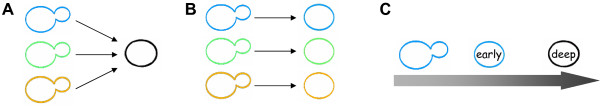
**(A) Quiescence may be a unique cellular state that results from a dedicated program committing cells to quiescence**. (B) Quiescence may vary depending on cell's history or (C) depending on the time spent in quiescence.

Quiescence is the most common cellular state on earth. While it is relatively easy to describe a proliferating cell, defining a quiescent cell is rather difficult. A commonly accepted, yet highly operational, definition of quiescence is "a reversible absence of proliferation". Consequently, a cell that is not dividing but eventually will when conditions become appropriate, is considered as a *bona fide *quiescent cell. But this definition is rather vague and probably encompasses various cellular situations. Therefore, instead of a single quiescent state, one can imagine that there may be distinct quiescent states depending on the cell's history before entry into quiescence, and/or depending on the time spent in quiescence (early quiescence, deep quiescence..., see Figure 1). This raises the delicate question of the existence of a quiescence "program". In other words, does quiescence result from a dedicated gene expression pattern that commits cells to the quiescent state or is quiescence an ultimate form of slow growth which would be a passive consequence of a cell's adaptation to unfavorable external conditions?

One major problem for studying quiescence comes from the fact that in multi-cellular organisms, environmental signals that control quiescence emanate from the entire organism - conditions that are difficult to reproduce in a lab. By contrast, in single cell eukaryotes like budding yeast, quiescence entry and exit are solely conditioned by nutrient availability. Using this model, the nature of proliferation/quiescence transitions has recently been revisited.

## Discussion

### Quiescence entry: a diversity of adaptations

Budding yeast is THE model in which genetics has proven its power, and not surprisingly, using this organism, several genetic approaches have been developed in order to identify a dedicated mechanism that drives cells into quiescence. Many mutants were found to be specifically sensitive to one nutrient limitation [1-5], but only very few of them died upon all the starvation conditions tested [1,2]. At the present time, it is not known whether these mutants fail to enter, maintain or exit quiescence. Further, the genetic background of the yeast strain used for the screen, and in particular the auxotrophic markers, greatly influences the final result [1], thus complicating the interpretation of genetic approaches for the study of quiescence.

Global transcriptional analyses have demonstrated that gene expression patterns greatly vary depending on cell's history. In a very elegant study, Coller and co-workers, have clearly shown that in fibroblasts forced to enter quiescence by different routes, the gene expression profiles, while very different in the early days of quiescence, converge toward a common signature after several days spent in quiescence [6]. This raises the possibility of the existence of different quiescent "states" depending on the time spent non-proliferating. It is not yet known whether early and deep quiescence notions are relevant in budding yeast. Nevertheless, in this organism it has clearly been shown that the overall mRNA profile was very different depending on the limiting nutrient. In fact, only a small set of genes are similarly regulated upon exhaustion of carbon, nitrogen, sulfur or phosphorus [2,7,8]. Therefore transcriptome data do not support the existence of a unique quiescent gene expression program that would commit yeast cells to the quiescent state. This assumption is further sustained by metabolomic studies revealing that none of the metabolic compounds measured showed a quiescence-specific behavior over all starvation conditions. Instead, the observed metabolic signatures were rather specific for the missing nutrient [1,9]. This strongly argues for the existence of different quiescent states depending on the history of the cell and reinforces the idea that quiescence might not be a committed state but rather an extreme manifestation of slow growth [1].

In fact, gene expression profiles of very slowly growing cells that are limited for one specific nutrient closely approximates the expression profile found in cells rendered quiescent by the total exhaustion of the same nutrient [1,8,10]. Moreover, it has been known for years that whatever the starvation conditions, quiescent cells acquire resistance to several stresses due to a modification of their cell wall [4]. Yet the same properties can be found in a population of extremely slow-growing cells in chemostat [1,11-14]. Additionally, upon carbon source exhaustion, a proportion of cells become denser [15], but again, this characteristic is apparently not specific of quiescent cells [16] and is probably due to the storage of specific carbohydrates that can occur in actively dividing cells during the oxidative phase of the metabolic cycle [17]. Finally, the idea of quiescence being an extreme form of slow growth is totally compatible with the fact that when nutrients are becoming scarce, or when protein synthesis is inhibited, the G1 phase of the cell cycle lengthens [18,19]. Therefore, the fact that in budding yeast, quiescence entry occurs preferentially in G1 could simply be the passive result of the metabolic slowdown.

### Quiescence entry: a commitment to a specific cellular organization

As presented above, there are several pieces of evidence for quiescence entry being an adaptative transition to a paroxystic form of slow growth. However, other arguments, listed below, rather point to a real commitment to quiescence.

We have recently shown that while quiescence entry and G1 arrest are generally concomitant in budding yeast, an arrest in G1 is neither necessary nor sufficient for quiescence establishment [20]. Furthermore, quiescent cells and G1 arrested cells have very different transcription profiles [5,21,22]. Besides, when auxotrophic strains are starved for the metabolite they are unable to synthesize, cells do not uniformly arrest in G1, yet they face starvation by modifying their gene expression and metabolism [7]. Importantly this response is not sufficient for entering quiescence and these cells ultimately die massively [23]. Interestingly, cell death can be rescued by the inactivation of proteins that have been implicated in nutrient sensing and quiescence establishment (TOR, Sch9...) [23]. Therefore *bona fide *quiescence entry needs something more than a simple adaptative slow down of growth.

A puzzling observation made more than 30 years ago by Lillie and Pringle reports that yeast cells start to synthesize glycogen when half of the initial glucose is consumed, whatever the initial concentration [24]. Another striking feature is that upon carbon source exhaustion, the last division may not be an "ordinary division" since a significant proportion of bi-budded cells has been observed [25]. Consistently, quiescent cell populations contain more than the proportion of 50% daughter cells expected from regular division [15]. Therefore it seems that cells somehow actively anticipate the fact that they will soon be starved.

Additionally, we have shown that upon carbon starvation yeast assemble specific cellular structures: Actin Bodies and Proteasome Storage Granules (PSGs). These structures respectively result from the reorganization of the actin cytoskeleton and the re-localization of the proteasome from the nucleus to the cytoplasm [26,27]. These structures have never been observed in dividing cells, and, under specific conditions, can be assembled independently of G1 [20]. This indicates again that quiescence entry is not subjugated to cell cycle regulation. Importantly, in budding yeast, both Actin Bodies and PSGs do assemble upon abrupt transfer of dividing cells into water, a condition that is probably closer to what may happen in the wild. Furthermore, studies from the Marcotte and Werner-Washburne groups have shown that upon carbon source exhaustion, several proteins re-localize, a majority of them being metabolic enzymes [28-30]. Therefore, at the ultra-structural level, cells are committed to the quiescent state. Undoubtedly, a sole proliferation arrest, even in G1, is not enough to trigger Actin Body formation. Further, the utilization of cell cycle mutants has revealed that quiescence entry can be triggered several hours after the proliferation arrest by carbon source exhaustion [20]. Thus proliferation arrest and quiescence entry are not necessarily concomitant.

### Quiescence exit: a metabolic switch?

Once committed to quiescence, cells need to survive and be able to maintain the capacity to re-proliferate. These two processes are poorly understood. Another layer of complexity is added by the fact that in budding yeast, mechanisms involved in transitions between quiescence and senescence or cell death are poorly understood. However, some light has recently been shed on the transition between quiescence and re-proliferation. Indeed, we found that a small proportion of cells can enter quiescence in another cell cycle phase than G1. Importantly, these cells can exit quiescence and give rise to progeny [20]. Therefore, like quiescence entry, quiescence exit is independent of G1.

Additionally, several studies have pointed to the fact that quiescent yeast cells seem to be somehow "prepared" to re-enter the proliferation state as fast as possible. Their transcription machinery is poised to specific gene promoters, ready to fire, and a burst of specific mRNA occurs in less than 5 minutes after nutrient addition [5,22]. Further, they make spatially arranged reserves that can be efficiently mobilized upon re-feeding. For example, actin is stored as Actin Bodies and long term polarity cues remain at one cell pole, both allowing to efficiently resume polarized cell growth [31,32]. Such a preparation strongly supports the idea of a quiescence program that "organizes" cells to be ready to proliferate as fast as possible upon nutrient replenishment.

Finally, the first committed step of quiescence exit does not require *de novo *protein synthesis. Indeed, structures found in quiescent cells can be readily mobilized upon nutrient re-feeding even in the presence of cycloheximide [26,27,29]. Consistently, the "mobilization signal" is rather metabolic and can be triggered by transferring quiescent cells into a glucose containing solution [20,33,34]. In fact, we have shown that glucose has to be metabolized at least into pyruvate to trigger Actin Bodies mobilization [20]. This suggests that a small molecule, yet to be identified, may act either directly on quiescent cell structures, or via a cascade leading to a post-translational modification of a key protein that will in turn cause the mobilization of quiescent cells structures. Importantly, this "mobilization signal" is not sufficient to trigger cells re-proliferation. Therefore, like proliferation exit and quiescence entry, quiescence exit and re-entry into proliferation can be uncoupled, but such a decoupling ultimately leads to cell death [33].

## Conclusion

In yeast, as in fibroblasts [6], a sole proliferation arrest is not sufficient to trigger quiescence entry and quiescent cells are more than just an ultimate form of slow growing G1 cells. Indeed, their commitment to a new cellular state is revealed by their re-organization at the ultra-structural level. Yet, recent evidence strongly supports the existence of various quiescent states depending on the cell's history. Therefore, quiescence could be the converging result of both an adaptive process allowing cells to cope with adversity and an active preparation to efficiently resume proliferation. Consequently the opposition between a quiescence-specific genetic program and an adaptation towards an ultimate form of slow growth might not be relevant. What is clear is that there is no need for subservience of quiescence entry and exit to cell cycle regulation. We rather propose that the metabolic status is a prevailing determinant for both quiescence entry and exit.

## Abbreviations

PSG: Proteasome Storage Granules;

## Competing interests

The authors declare that they have no competing interests.

## Authors' contributions

I.S. and B.D.-F wrote the manuscript. The authors read and approved the final manuscript.
